# Understanding inequities in health and health systems in Latin America and the Caribbean: a thematic series

**DOI:** 10.1186/s12939-021-01426-1

**Published:** 2021-04-06

**Authors:** Ana Lorena Ruano, Daniela Rodríguez, Pablo Gaitán Rossi, Daniel Maceira

**Affiliations:** 1 Center for International Health, Department of Global Public Health and Primary Care, University of Bergen, Bergen, Norway; 2 Center for the Study of Equity and Governance in Health Systems (CEGSS), Guatemala, Guatemala; 3 Department of International Health, Health Systems Division, Bloomberg School of Public Health Johns Hopkins, Baltimore, USA; 4 Instituto de Investigaciones para el Desarrollo con Equidad (EQUIDE), Universidad Iberoamericana, Ciudad de México, Mexico; 5 Center for the Study of State and Society (CEDES), Buenos Aires, Argentina

## Abstract

Latin America, with its culturally and ethnically diverse populations, its burgeoning economies, high levels of violence, growing political instability, and its striking levels of inequality, is a region that is difficult to define and to understand. The region’s health systems are deeply fragmented and segmented, which poses great challenges related to the provision of quality of care and overall equity levels in health and in Latin American society at large. Market, social, and political forces continue to push towards the poorly regulated privatization of public health care in many countries within the region, in detriment of public healthcare services where management capacities are limited.

In this first collection of papers, we showcase how the region has tackled, with different levels of success, the incorporation of innovative health system reforms aimed at strengthening governance, participation, and the response to the growing epidemiological and demographic demands of its diverse population. We are delighted that this Special Collection will remain open to house future papers from Latin America and the Caribbean. The region has important experiences and lessons to share with the world. We look forward to learning more about how researchers and practitioners continue to experiment and innovate in their struggle to reach equity in health for all. This thematic series is a platform where the region’s lessons and approaches can be shared with the global community of Health Policy and Systems Researchers.

Latin America, with its culturally and ethnically diverse populations, its burgeoning economies, high levels of violence, growing political instability, and its striking levels of inequality, is a region that is difficult to define and to understand. Latin American health systems are deeply fragmented and segmented, which poses great challenges related to the provision of quality of care and overall equity levels in health and in Latin American society at large. This is the result of complex historical, political, and economic country and sub-regional contexts, that limit the region’s capacity to provide equitable access to quality healthcare and public health services and to ensure the right to health of its populations, a right enshrined in most of its countries’ progressive legal frameworks. These inequities are also evident in the region’s financially strained health systems that are unable to guarantee quality care to many vulnerable or traditionally excluded populations, particularly women and girls, the LGBTQ+ community, indigenous peoples, ethnic minorities, and migrants and refugees.

Market, social, and political forces continue to push towards the poorly regulated privatization of public health care in many countries within the region, in detriment of public healthcare services where management capacities are limited. The first article collection in this thematic series showcases how the Latin American region has tackled, with different levels of success, the incorporation of innovative health system reforms aimed at strengthening governance, participation, and the response to the growing epidemiological and demographic demands of its diverse population. Within these packages of reforms were those aimed at decentralizing and restructuring public healthcare provision during the 1990s [1]. As a result of these and other policies, Palacios, Espinola and Rojas Roque [2] found that the Argentine health system shows pro-rich inequality in the use of public services, and that this is fueled by the inequitable distribution of access to the social security institute and to the private healthcare sub-system. Bernales-Baksai [3] addresses the issue in Chile and Uruguay, countries with seemingly strong health system in the region, and shows how their policy architectures tackle segmentation. She found that equity gaps continue to exist and that, as González & Triunfo point out in their study of horizontal equity in Uruguay [4], these are fueled by historical and structural issues that hinder the development of solidarity between social classes and push higher income families towards private care.

Health system reforms in the region need to address changing population and epidemiological profiles. For example, the paper by Santamaría-Ulloa and Montero-López [5] projects that the prevalence of diabetes in the older adult population in Costa Rica will double in 13 years with an estimated loss of 7 months of life. Without adaptations to the healthcare system, the health and economic consequences will be substantial.

The Americas are home to almost forty million indigenous people [6] and they represent a significant proportion of the population in countries like Mexico, Guatemala, Colombia, Bolivia, Peru, Chile, and Brazil [7]. However, indigenous peoples, as one of the most excluded population groups in the region, have worse health outcomes than settler descendants and have been historically excluded from engaging in policy development. Samuel, Flores and Frisancho [8] argue that the exclusion of indigenous peoples hinders the region’s path towards Universal Health Coverage (UHC). They found that progressive legal frameworks and increasing health budgets are not enough to guarantee access to quality care and posit that it is only through the development and implementation of more inclusive accountability mechanisms that many marginalized populations in Peru and Guatemala will be able to access the type of care that can protect them from financial hardship. Linked to this call for accountability, Ferdinand et al. [9] compared the engagement of indigenous peoples in Chile and Brazil with Australia and New Zealand, and found that the participation of these ethnic and cultural groups has been shaped by the way countries adopt international agreements and how they are reflected in national policy and legal frameworks. However necessary these frameworks are, they are not enough to overcome inequities, and multi-level approaches that improve cohesion are necessary. Garnelo et al’s [10] work on the limitations of fluvial mobile units to reach riverside indigenous communities highlights how strategies aimed at expanding the scope of primary healthcare in the region are insufficient if they do not actively address the core issues of inadequate and insufficient investments to reach indigenous peoples in the places where they live and work. Further, the health system will continue to underuse valuable resources like community health workers at the expense of improving health equitably.

Sexual and reproductive health continue to be a significant challenge in the region, reflecting the exclusion of women, girls, and people belonging to the LBGTQ+ community. In their study of maternal mortality in Colombia, Rivillas, Devia Rodríguez and Ingabire [11] found that fragmented health systems yield differential outcomes in maternal mortality and thus increases inequality. Tackling this entails reviewing and adjusting the country’s public and subsidized health insurance scheme to deal with the growing exclusion of poor women from marginal areas. Actively addressing exclusion requires going beyond health system financing to develop policies aimed at including afro-descendants, indigenous peoples, and others who live in poverty and/or are geographically excluded from accessing health facilities, while addressing health worker capacities and provider incentives. Obstetric violence continues to be a central issue in the region: Roldán, Grajeda & Pérez found that Cesarean section rates in Guatemala were high and above international recommendations, with the bulk of these procedures being performed in shorter women [12]. In the country, indigenous and poor women tend to be much shorter than their non-indigenous counterparts, which points towards the overuse of a medical procedure in a traditionally marginalized population group that already faces compounding factors that limit the use of their agency when it comes to deciding how and when to give birth. Finally, Calderon Jaramillo et al. [13] present an innovative article looking at how transgender people access health services in Colombia, and how health policies address their specific needs. They find that, in order to meet the needs of this population, health systems must address the structural barriers (e.g., costs, lack of insurance), service delivery discrimination (e.g., stigmatization, abuse from healthcare providers) and rights-based clinical services (e.g., endocrinology and surgical services) unique to this population.

Over the last 20 years, countries in Latin America and the Caribbean have been experimenting with different methods and approaches to improve health system performance. Conditional cash transfers (CCTs) have been used extensively in the region as a way to address the economic and social exclusion of the poor and marginalized [14]. In their paper about CCTs, Poirier [15] found that Peru and Ecuador have been able to more effectively target their poorest populations, and that this is possible using universal and geographic targeting. Through the use of simple prediction models, CCT programs can be better implemented and help the push of ‘leaving no one behind’ while also controlling for clientelism, a common fear around CCTs in the region. Moreover, high-quality methodologies can help assess the impact of health policies with greater precision. The paper by Tomie Ivata Bernal et al. [16] illustrates how the use of a small-area prevalence estimation identified that the reduction in tobacco prevalence in Brazil was more common in high-income areas than it was in the lower-income areas. The ability to replicate these approaches to use more granular information may foster better local policies.

This Special Collection begins to capture some of the diversity in experience, health systems and contextual dynamics in Latin America, but key gaps remain. Voices from the Caribbean are not yet here despite, or perhaps because of, the historical complexities that the sub-region faces. How these countries will tackle looming challenges arising from the threat of climate change to the island nations in this sub-region remains to be seen. Also missing is research and practice that delves into the lasting consequences of the region’s dictatorial and authoritarian regimes, civil wars, organized crime, and militia groups on health outcomes, the framing of health, the government’s social contract with its most underserved and vulnerable populations, and the politics of restitution for harms done. Additionally, we call for papers that study and analyze the Venezuelan refugee crisis, one of the largest of the world [17, 18]. This call opens opportunities to explore how Latin America, a region with ostensibly more stability and economic capacity, has coped with an influx of migrants both in terms of addressing their health needs and how migrant human resources for health are integrated into local health systems. Documenting the impact that this humanitarian crisis has had on Venezuela and on the countries receiving migrants leaving the country is key to improving their access to quality healthcare services that respond to their needs.

The region has a rich history around community advocacy and participatory approaches, which we hope will be reflected in future submission to our thematic series. We also look forward to papers on adolescent health as well as on issues around the provision of mental health services, and to critical analyses of how the region is coping with environmental health, demographic change, and ageing. Lastly, we would be remiss in not pointing out that the COVID-19 crisis has laid bare the deep and vast wounds that inequity and inequality have left in the region. Future research will surely capture the differential impacts of a public health response that draws on evidence and science as well as coping mechanisms of an overwhelmed public sector in a fragmented health system.

We are delighted that this Special Collection will remain open to house future papers from Latin America and the Caribbean. The region has important experiences and lessons to share with the world. We look forward to learning more about how researchers and practitioners continue to experiment and innovate in their struggle to reach equity in health for all. This thematic series is a platform where the region’s lessons and approaches can be shared with the global community of Health Policy and Systems Researchers. The next Health Systems Symposium will be held in Bogota in 2022, and it is our distinct hope that this is a first step towards a truly global debate that builds on our challenges, lessons learned, and innovations.

## References

[1] Vazquez ML, Siqueira E, Kruze I, Da Silva A, Leite IC (2002). The reform process and social participation in health in Latin America. Gac Sanit.

[2] Palacios A, Espinola N, Rojas-Roque C (2020). Need and inequality in the use of health care services in a fragmented and decentralized health system: evidence for Argentina. Int J Equity Health.

[3] Bernales-Baksai P (2020). Tackling segmentation to advance universal health coverage: analysis of policy architectures of health care in Chile and Uruguay. Int J Equity Health.

[4] González C, Triunfo P (2020). Horizontal inequity in the use and access to health care in Uruguay. Int J Equity Health.

[5] Santamaría-Ulloa C, Montero-López M (2020). Projected impact of diabetes on the Costa Rican healthcare system. Int J Equity Health.

[6] Del Popolo F, Jaspers D (2014). Guaranteeing indigenous people’s rights in Latin America. Progress in the past decade and remaining challenges. Summary.

[7] Montenegro RA, Stephens C (2006). Indigenous health in Latin America and the Caribbean. Lancet.

[8] Samuel J, Flores W, Frisancho A (2020). Social exclusion and universal health coverage: health care rights and citizen-led accountability in Guatemala and Peru. Int J Equity Health.

[9] Ferdinand A, Lambert M, Trad L, Pedrana L, Paradies Y, Kelaher M (2020). Indigenous engagement in health: lessons from Brazil, Chile, Australia and New Zealand. Int J Equity Health.

[10] Garnelo L, Parente RCP, Puchiarelli MLR, Correia PC, Torres MV, Herkrath FJ (2020). Barriers to access and organization of primary health care services for rural riverside populations in the Amazon. Int J Equity Health.

[11] Rivillas JC, Devia-Rodriguez R, Ingabire M-G (2020). Measuring socioeconomic and health financing inequality in maternal mortality in Colombia: a mixed methods approach. Int J Equity Health.

[12] Roldán E, Grajeda LM, Pérez W (2020). Maternal height associated with cesarean section. A cross-sectional study using the 2014–2015 national maternal-child health survey in Guatemala. Int J Equity Health.

[13] Calderón-Jaramillo M, Mendoza Á, Acevedo N, Forero-Martínez LJ, Sánchez SM, Rivillas-García JC (2020). How to adapt sexual and reproductive health services to the needs and circumstances of trans people— a qualitative study in Colombia. Int J Equity Health.

[14] Osorio Gonnet C (2019). A comparative analysis of the adoption of conditional cash transfers programs in Latin America. J Comp Policy Anal Res Pract.

[15] Poirier MJ (2020). Geographic targeting and normative frames: revisiting the equity of conditional cash transfer program distribution in Bolivia, Colombia, Ecuador, and Peru. Int J Equity Health.

[16] Bernal RTI, de Carvalho QH, Pell JP, Leyland AH, Dundas R, Barreto ML, Malta DC (2020). A methodology for small area prevalence estimation based on survey data. Int J Equity Health.

[17] R4V. Coordination platform for refugees and migrants from Venezuela https://r4v.info/en/situations/platform: response for Venezuelans; 2020 [updated 5 Oct 2020.

[18] Bahar D, Dooley M. Venezuela refugee crisis to become the largest and most underfunded in modern history https://www.brookings.edu/blog/up-front/2019/12/09/venezuela-refugee-crisis-to-become-the-largest-and-most-underfunded-in-modern-history/: Brookings; 2019 [updated 9 Dec 2019.

